# Supplementary material for: “Grafting-from” and “Grafting-to” Poly(N-isopropyl acrylamide) Functionalization of Glass for DNA Biosensors with Improved Properties
Source: Polymers (Basel). 2024 Oct 11;16(20):2873. doi: 10.3390/polym16202873 (PMC11510813; doi:10.3390/polym16202873)
Supplement: Supplementary file 1 [file polymers-16-02873-s001.zip › polymers-3238983-supplementary.pdf]

Supporting information

"Grafting-from" and "Grafting-to" Poly(N-isopropyl acrylamide) Functionalization of Glass for DNA Biosensors with Improved Properties

Pauline Skigin[a,†], Perrine Robin[a,†], Alireza Kavand [a], Mounir Mensi [b] and Sandrine Gerber-Lemaire [a,*]

[a] Institute of Chemical Sciences and Engineering, Group for Functionalized Biomaterials, Ecole Polytechnique Fédérale de Lausanne, EPFL SB ISIC SCI-SB-SG, Station 6, CH-1015 Lausanne, Switzerland.
[b] ISIC-XRDSAP, EPFL Valais-Wallis, Rue de l'Industrie 17, CH-1951, Sion, Switzerland; mounir.mensi@epfl.ch
* Correspondence: sandrine.gerber@epfl.ch
† These authors contributed equally.

Table of contents

1. General **2**

1.1 Materials *2*

1.2 Instrumentation and Characterization *2*

2. In-solution Study **3**

2.1 PET-RAFT Polymerization: A Kinetic Study *3*

2.2 Post-Polymerization Modifications *4*

2.3 Analysis of the Polymers by GPC *7*

2.4 LCST Measurements in Solution *7*

3. Surface Functionalization and Evaluation **8**

3.1 Synthesis of Silanization Reagent *8*

3.2 DNA Hybridization Density *9*

3.3 Water Contact Angle *9*

3.4 Protein Fouling Assey *9*

1. General

1.1. Materials

The following were used: N-isopropylacrylamide (NIPAM, 99%, Sigma-Aldrich, Darmstadt, Germany), (2-dodecylthiocarbonothioylthio)-2-methylpropionic acid (DTTMP, 98%, TCI, Zwijndrecht, Belgium), 2,2'-Azobis(2-methylpropionitrile), 2-(azo(1-cyano-1-methylethyl))-2-methylpropane nitrilerecrystallized in ethanol prior to use (AIBN, 98%, Fluka, Buchs, Switzerland), sodium hydrosulfite ($Na_2S_2O_4$, 82%, Sigma Aldrich, Darmstadt, Germany), propylamine (99%, Sigma Aldrich, Darmstadt, Germany,), triethylamine anhydrous (TEA, 99%, Fluorochem, Hadfield, United Kingdom), Eosin Y (Acros Organics, Geel, Belgium), 1,3,5-trioxane (99%, Sigma-Aldrich, Darmstadt, Germany) TCEP·HCl (Thermo Scientific, Waltham, MA, USA), succinic anhydride (95%, TCI, Zwijndrecht, Belgium), 1-(3-dimethylaminopropyl)-3-ethylcarbodiimide hydrochloride (EDC·HCl, 98%, Acros Organics, Geel, Belgium), 1-hydroxybenzotriazole hydrate (HOBt, 97%, Sigma Aldrich, Darmstadt, Germany,), 3-aminopropyltriethoxysilane (APTES, 99%, Acros Organics, Geel, Belgium), dichloromethane (DCM, 99.9% ACS Reagent, Acros Organics, Geel, Belgium), dimethyl sulfoxide (DMSO, 99.7 % extra dry, Thermo Scientific, Waltham, MA, USA), acetonitrile (ACS reagent, Supelco, Bellefonte, Pennsylvania, USA), toluene (99.85% extra dry, Thermo Scientific, Waltham, MA, USA), tetrahydrofuran (THF, 99.8% anhydrous, Thermo Scientific, Waltham, MA, USA), and 1,4- dioxane (99.8% extra dry, Acros Organics, Geel, Belgium), diethyl ether (99.5%, Riedel-de Haën, Seelze, Germany).

Borosilicate substrates (10 mm × 10 mm × 0.5 mm, ISO class 5 clean room production) were purchased from SCHOTT AG. Single-strand oligonucleotides (ssDNA) were purchased from GenScript and diluted to 100 µM using MilliQ water. The following sequences of Zika ZAS1 were used as the probe and Cy3-tagged complementary strand,respectively: 5' NH_2-C6-GCCGCCTCGCCCATCTCAACCC 3' and 5' Cy3-GGGTTGAGATGGGCGAGGCGGC 3'. Buffer solutions were prepared with MilliQ water. MES 0.5 M pH 6 was prepared with MES hydrate (99.5%, Sigma-Aldrich); phosphate-buffered saline (PBS) 0.1X was prepared by diluting commercial PBS pH 7.4 1X (Gibco); saline-sodium citrate (SSC) 4X was prepared by diluting commercial SSC buffer 20X (Thermo Scientific, Waltham, MA, USA); Tris 0.1M was prepared from Tris-HCl pH 8.0 1M (Thermo Scientific, Waltham, MA, USA). Tween 20 (molecular biology grade, Serva, Heidelberg, Germany) was used to prepare Tween 0.1%. Albumin from bovine serum and fluorescein conjugate (AF488-FITC) were purchased from Invitrogen by Thermo Fischer Scientific (Waltham, MA, USA).

1.2. Instrumentation and Characterization

Photopolymerization reactions were performed in a SynLED Parallel Photoreactor (Sigma-Aldrich, Darmstadt, Germany). Centrifugation steps were carried out using an Allegra X-30R centrifuge (Beckman Coulter Life Sciences, Brea, CA, USA). Purification by dialysis was performed with Spectra/Por 7 dialysis membranes (MWCO: 1 kD) in distilled H_2O, and the water was changed every three hours for two days. NMR spectra were recorded on a Bruker ACIII-400 spectrometer, and the chemical shifts are hereby reported in ppm relative to the deuterated chloroform resonance. The peak integrals of polymeric compounds are listed according to their monomeric counterpart. The LCST of free polymers was studied by measuring the absorbance of 5 mg/mL solutions at 500 nm, using an Agilent

Cary 60 UV-Vis spectrophotometer equipped with a temperature probe. During the surface functionalization steps, test tubes were incubated using a MSC100 Cooling Thermoshaker incubator. Water contact angles were measured using an Easy Drop from Krüss. X-ray photoelectron spectroscopy (XPS) measurements were carried out on Axis Supra from Kratos Analytical, using the monochromated Ka X-ray line of an aluminum anode. Charge neutralization was used, and the spectra were referenced at 284.8eV using the CC/CH component of the C1s manifold. As XPS is known to potentially damage sensitive species, S 2p and P 2p orbitals were measured first. Fluorescence intensity measurements of the solutions were performed on a BioTek Synergy H1 microplate reader. Fluorescence intensity measurements of the surfaces were performed with a Leica DM5500 Upright Microscope.

2. In-solution Study

2.1. PET-RAFT Polymerization: A Kinetic Study

NIPAM (50 mg, 0.390 mmol) and DTTMP (4.7 mg, 0.013 mmol) were dissolved in 1 mL of dry THF or DMSO. TEA (100 µL, 13 $mg.mL^{-1}$ in DMSO) and Eosin Y (100 µL, 9 $mg.mL^{-1}$ in DMSO) were added to reach a [30]:[1]:[0.01]:[1] molar ratio (NIPAM/DTTMP/Eosin Y/TEA). 1,3,5-Trioxane (13 mg) was added as a reference for NMR measurements, and the reaction mixture was degassed with a flux of argon for 10 minutes. Polymerization was initiated by placing the reaction in the photoreactor at 465-470 nm illumination and terminated by switching off the light source. ^{1}H NMR spectra were recorded at different time points: i) at t = 0 and t = 3 hours for the evaluation of the solvent effect; and ii) every hour from t = 0 to t = 5 hours for the kinetic study in DMSO. Integrals of the peaks highlighted in green (Figure S1) were used for the computation of monomer conversion.

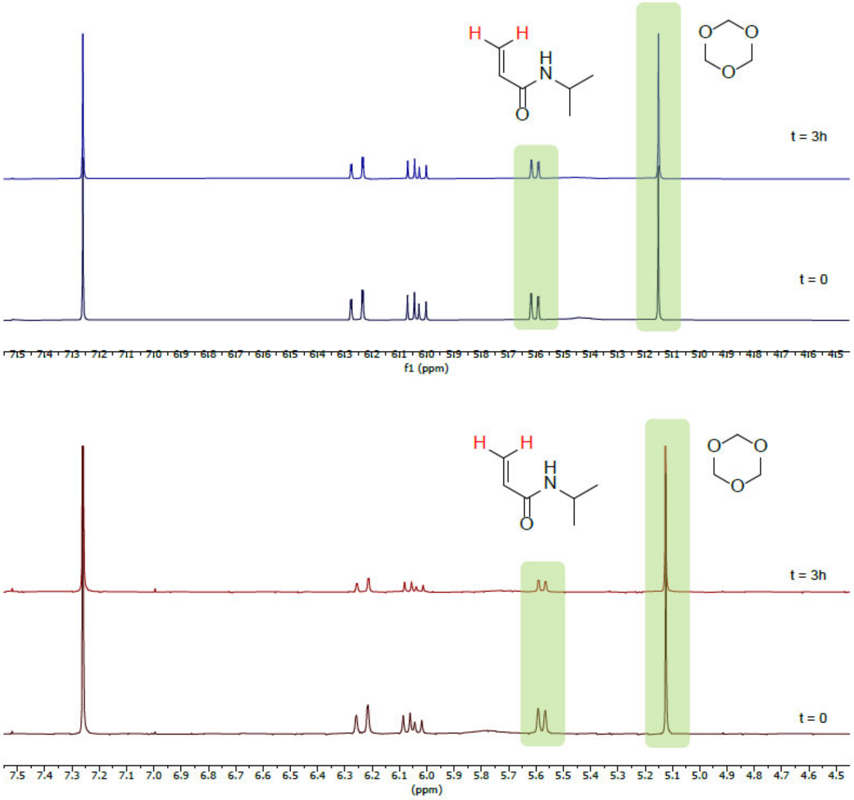

Figure S1. Monomer conversion study of PET-RAFT in THF (top) and DMSO (bottom), with highlighted monomer and 1,3,5-trioxane peaks used for conversion computation (green).

2.2. Post-Polymerization Modifications

Synthesis of PNIPAM-CTA via conventional RAFT polymerization. NIPAM (1000 mg, 7.86 mmol), DTTMP (94.7 mg, 0.26 mmol), and AIBN (8.6 mg, 0.05 mmol) were dissolved in dry THF (7 mL) to a [30]:[1]:[0.2] molar ratio (NIPAM/DTTMP/AIBN). 1,3,5-Trioxane (5 to 10 mg) was added to the solution, which was thoroughly degassed with a flux of argon for 20 minutes. The reaction mixture was stirred at 65°C for 18 hours and abruptly cooled to 0°C with an ice bath. The product was precipitated in cold diethyl ether and centrifuged (4700 rpm, 5 minutes), and the supernatant was removed. **PNIPAM-CTA** (753 mg, 0.23 mmol, 88%) was collected as a yellow solid and dried at room temperature under vacuum.

The following values were obtained: ^{1}H NMR (400 MHz, CDCl3) δ 4.00 (br, 1H × 29 monomers, NH-C*H*-(CH_3)$_2$), 2.15-2.07 (br, 2H × 29 monomers, CH-C*H*$_2$-S), 1.67-1.59 (br, 1H × 29 monomers, C*H*-CH_2-S), 1.30 (s, 6H, 2 × C*H*$_3$), 1.27-1.12 (br, 2 × C*H*$_3$ × 29 monomers,, plus 11 ×C*H*$_2$-CH2), and 0.86 (t, 3H, C*H*$_3$).

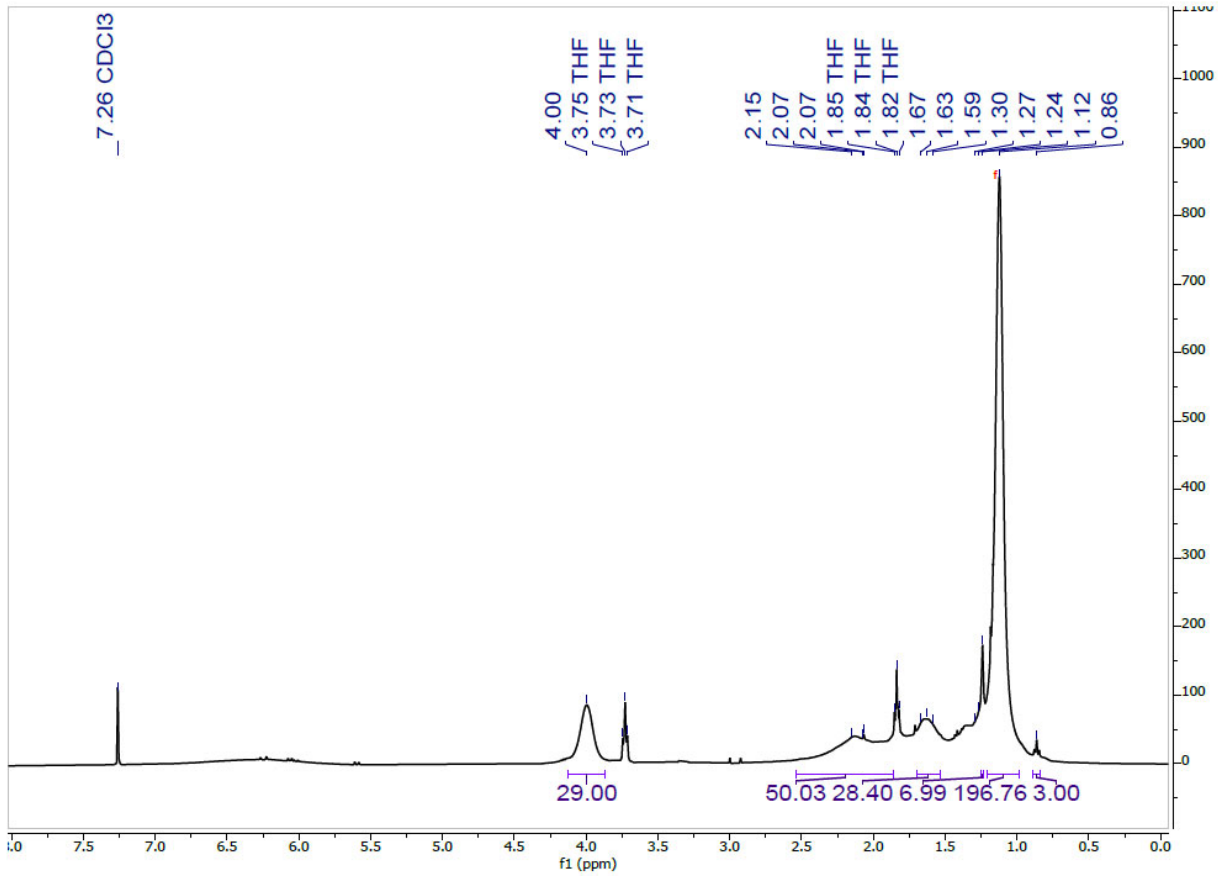

Figure S2. ^{1}H NMR of **PNIPAM-CTA** synthetized via conventional RAFT. The polymer is composed of 29 monomers.

Synthesis of PNIPAM-SH. PNIPAM-CTA (100 mg, 0.03 mmol, 1 eq.) was dissolved in dry THF (5 mL), to which a 0.6 M solution of $Na_2S_2O_4$ (10.5 mg in 100 µL distilled water, 0.06 mmol, 2 eq.) was added. After degassing the solution with a flux of argon for 20 minutes, propylamine (25 µL, 0.3 mmol, 10 eq.) was added and the reaction mixture stirred at room temperature for 4 hours under an inert atmosphere. The solvent was evaporated under reduced pressure and the crude product dissolved in dry THF (1 mL). The polymer was precipitated in cold diethyl ether (40 mL), centrifuged (4700 rpm, 5 minutes), and the supernatant was removed. **PNIPAM-SH** (89 mg, 0.029 mmol, 96%) was collected as a white solid and dried at room temperature under vacuum.

The following values were obtained: ^{1}H NMR (400 MHz, CDCl3) δ 4.00 (br, 1H × 29 monomers, NH-C*H*-(CH_3)$_2$), 2.13-1.76 (br, 2H × 29 monomers, CH-C*H*$_2$-S), 1.61 (br, 1H × 29 monomers, C*H*-CH_2-S), 1.24 (s, 2 × C*H*$_3$), and 1.12 (br, 2 × C*H*$_3$ × 29 monomers).

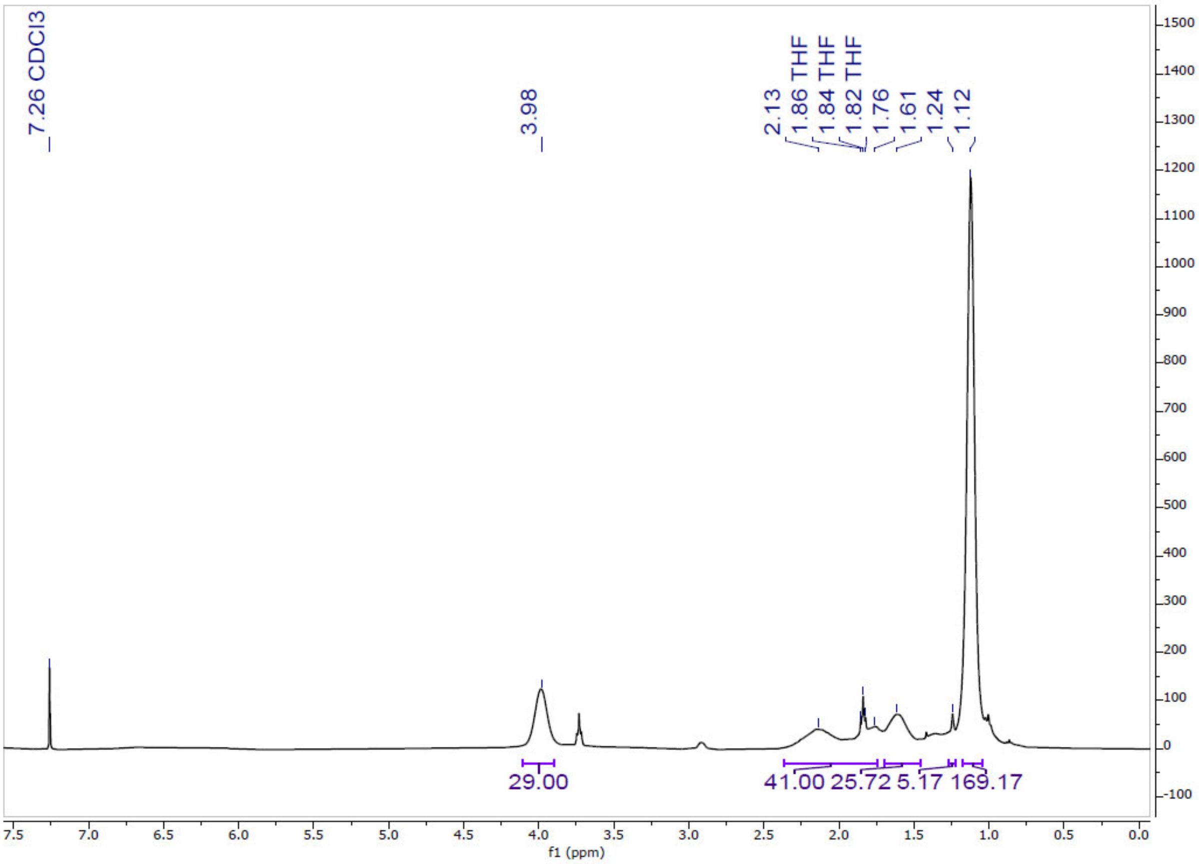

Figure S3. ^{1}H NMR of **PNIPAM-SH**. The polymer is composed of 29 monomers.

***Synthesis of* PNIPAM-COOH. PNIPAM-SH** (30 mg, 9.8 µmol, 1 eq.) and TCEP·HCl (4.3 mg, 15.0 µmol, 1.5 eq.) were dissolved in distilled water (3 mL) under an inert atmosphere. The reaction mixture was stirred at room temperature for 3 hours, and the solvent evaporated under reduced pressure. The crude product was dissolved in acetonitrile (10 mL) and filtered on a Büchner apparatus to remove the excess of TCEP·HCl. The filtrate was concentrated under reduced pressure. The residue was added to a solution of succinic anhydride (2 mg, 19.6 µmol, 2 eq.) in dry 1,4-dioxane (3 mL), under an inert atmosphere. The reaction mixture was stirred at room temperature overnight. The solvent was evaporated. The crude product was dissolved in distilled water (5 mL) and purified by dialysis. **PNIPAM-COOH** (9.4 mg, 3.0 µmol, 30%) was collected as a white solid.

The following were obtained: ^{1}H NMR (400 MHz, CDCl3) δ 3.98 (br, 1H × 29 monomers, NH-C*H*-(CH3)2), 2.96 (br, 2H, C*H*2 succinic), 2.75 (br, 2H, C*H*2 succinic), 2.17-1.81 (br, 2H × 29 monomers, CH-C*H*2-S), 1.64 (br, 1H × 29 monomers, C*H*-CH2-S), 1.25-1.03 (br, 2 × C*H*3 × 29 monomers plus 2 × C*H*3).

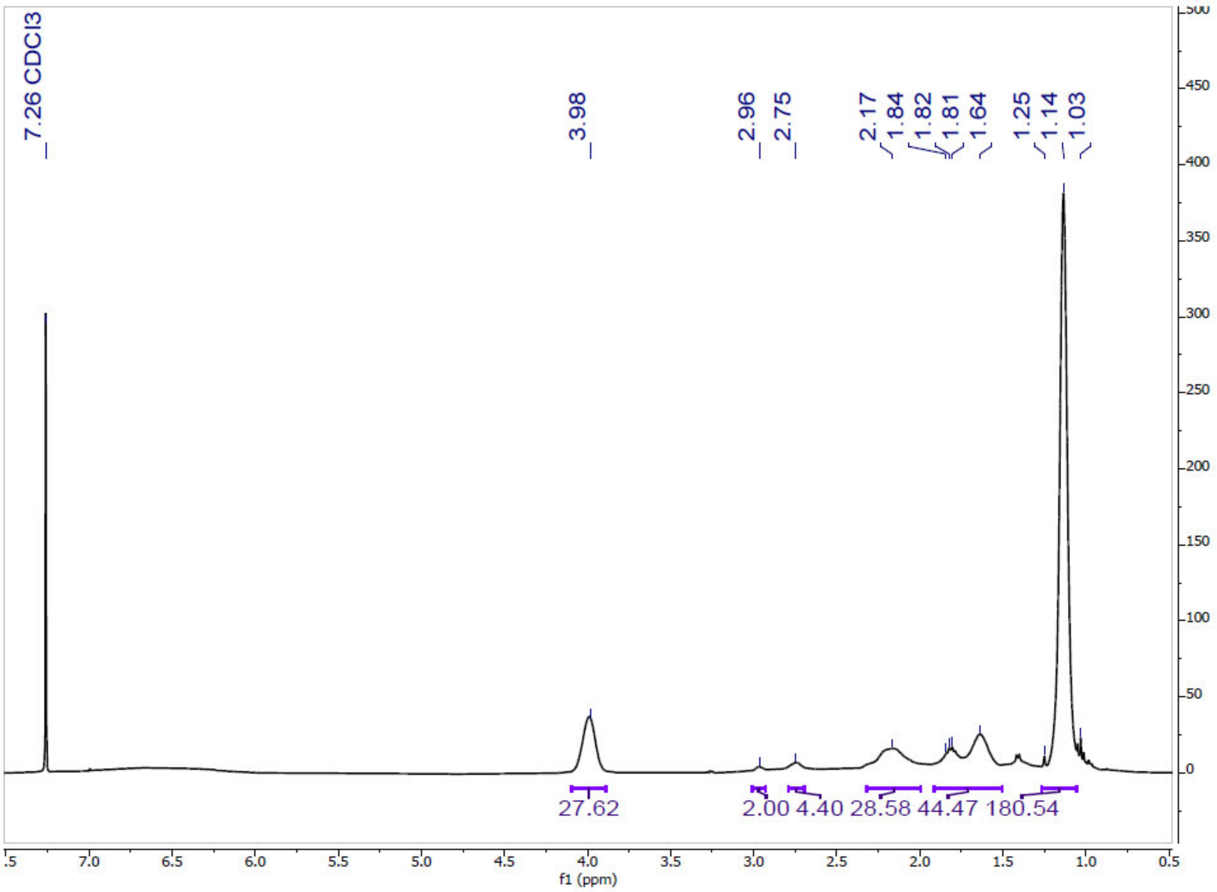

Figure S4. ^{1}H NMR of **PNIPAM-COOH**. The polymer is composed of 29 monomers.

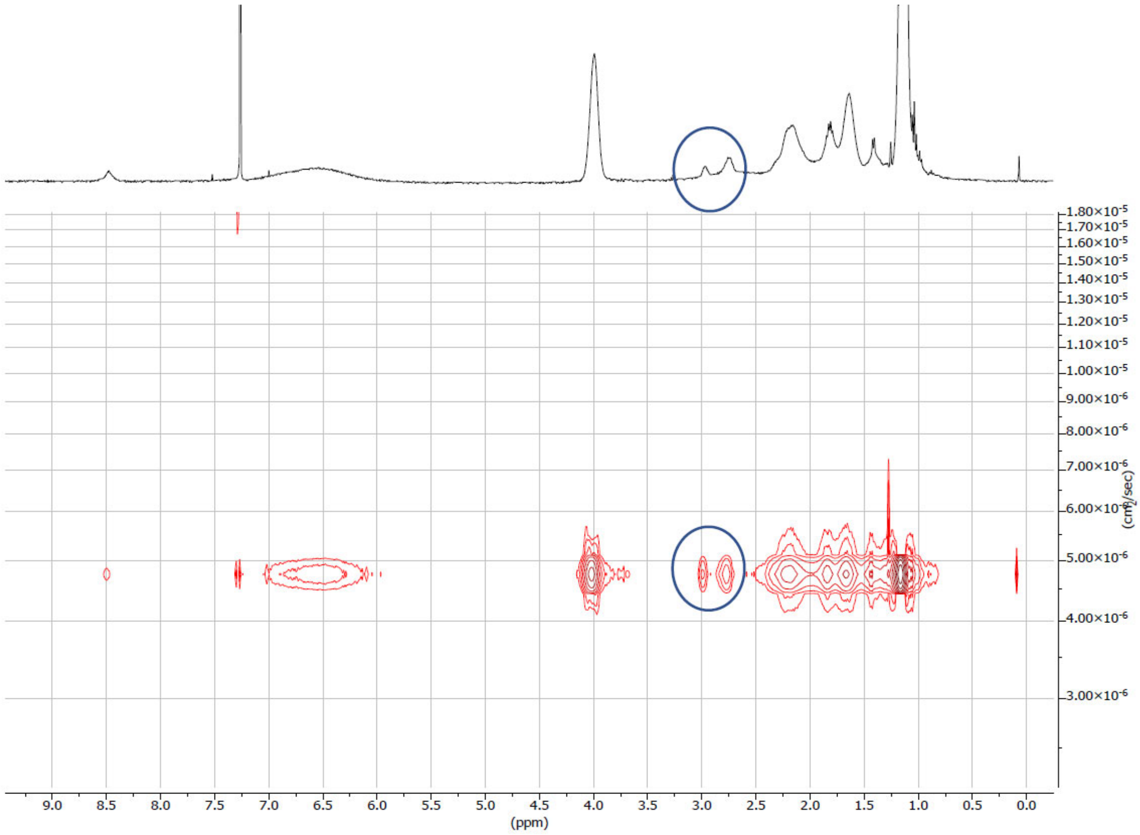

Figure S5. Two-dimensional DOSY NMR of **PNIPAM-COOH**. The two peaks corresponding to the succinic unit are highlighted.

2.3 *Analysis of the Polymers by GPC*

The molecular weight and polydispersity index (Đ = M_w/M_n) were determined by GPC, equipped with a PSS-Polymer SECcurity² system. The measurements were recorded at 70°C, using dimethylacetamide containing 0.1 wt% lithium bromide as the eluent.

Table S1. Molecular weight of polymer samples evaluated by gel-permeation chromatography.

	Polymer sample	Mn (g.mol^{-1})	Mw (g.mol^{-1})	PDI
conventional RAFT polymerization	*PNIPAM-CTA*	5319	7869	1.48
	PNIPAM-SH	4839	6540	1.35
	PNIPAM-COOH	6213	7762	1.25
PET-RAFT	Polymer from "grafting from" functionalization	3796	6555	1.73

2.4. *LCST Measurements in Solution*

The LCST of **PNIPAM-COOH** was evaluated by measuring the solution transmission in different media—MilliQ water, PBS 0.1X, and Tris 0.1 M at 5 mg·mL^{-1} at 500 nm. The LCST was computed as the threshold value at which the transmittance shifted below 50%.

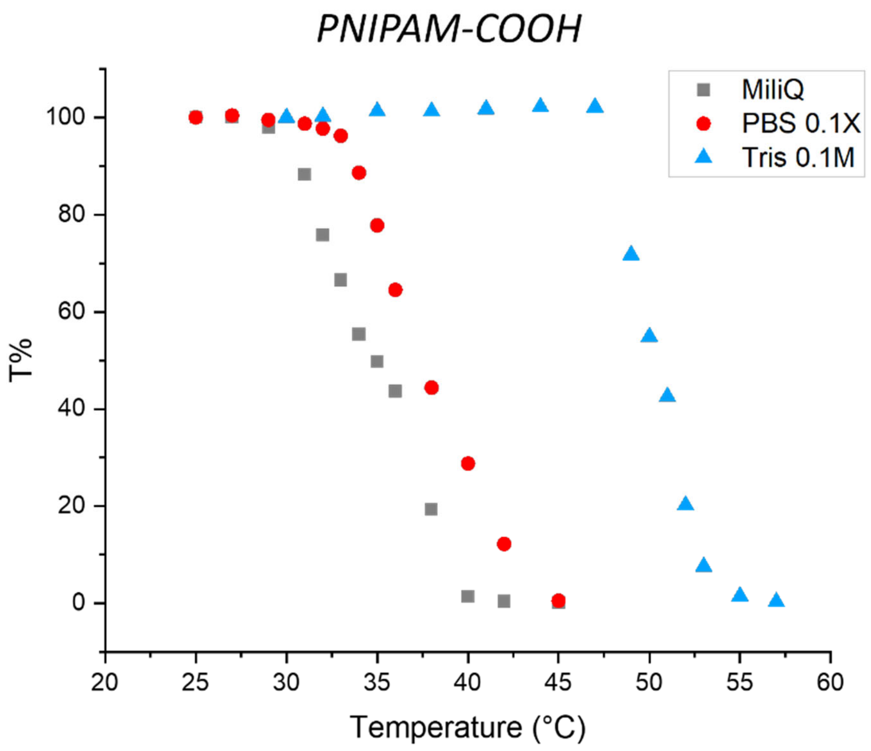

Figure S6. Transmittance of **PNIPAM-COOH** derivatives in MilliQ, PBS 0.1X, and Tris 0.1M media. The transmittance was measured by temperature-controlled UV-Vis spectrometry at 500 nm of polymer solutions at 5 mg·mL^{-1}. The buffer had a major impact on the thermoresponsive behavior. The LCST was 30-31°C in MiliQ, 33-34°C in PBS 0.1X, and 51-52 °C in Tris 0.1 Mfor **PNIPAM-COOH**.

3. Surface Functionalization and Evaluation

3.1. Synthesis of Silanization Reagent

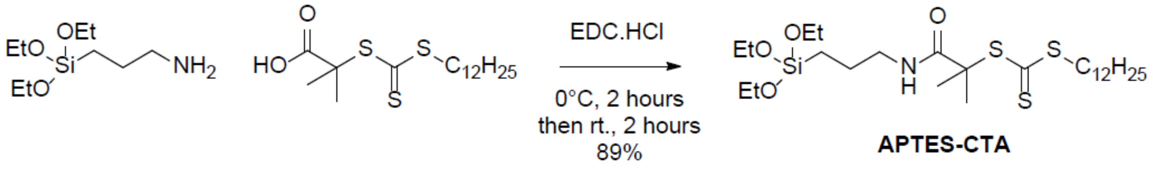

Scheme S1. Synthesis of **APTES-CTA**.

Synthesis of APTES-CTA. The synthesis of **APTES-CTA** was carried out following the procedures reported in the literature [1]. DTTMP (55 mg, 0.151 mmol, 1 eq.) and EDC·HCl (33 mg, 0.172 mmol, 1.1 eq.) were dissolved in dry DCM (5 mL) under an inert atmosphere, and the solution was cooled to 0°C. APTES (40 µL, 0.171 mmol, 1.1 eq.) was added, and the reaction mixture was stirred at 0°C for 2 hours and then for 2 hours at room temperature. The solvent was evaporated under reduced pressure, and the crude product was purified by flash column chromatography (hexane/ethyl acetate 4:1) to provide **APTES-CTA** (76 mg, 0.134 mmol, 89%) as a yellow oil.

The following were obtained: 1H NMR (400 MHz, CDCl3) δ 6.63-6.62 (t, 1H), 3.87-3.80 (q, 6H), 3.28-3.20 (m, 4H), 1.73-1.64 (m, 8H), 1.62-1.55 (p, 2H), 1.43-1.36 (m, 2H), 1.25-1.20 (m, 25H), 0.88-0.86 (t, 3H), 0.61-0.57 (t, 2H). HRMS (ESI/QTOF) m/z: [M + Na]+ Calculated for C26H53NNaO4S3Si+ 590.2798, Found 590.2794.

1. Li M, Fromel M, Ranaweera D, et al (2019) SI-PET-RAFT: Surface-Initiated Photoinduced Electron Transfer-Reversible Addition–Fragmentation Chain Transfer Polymerization. ACS Macro Lett 8:374–380. https://doi.org/10.1021/acsmacrolett.9b00089

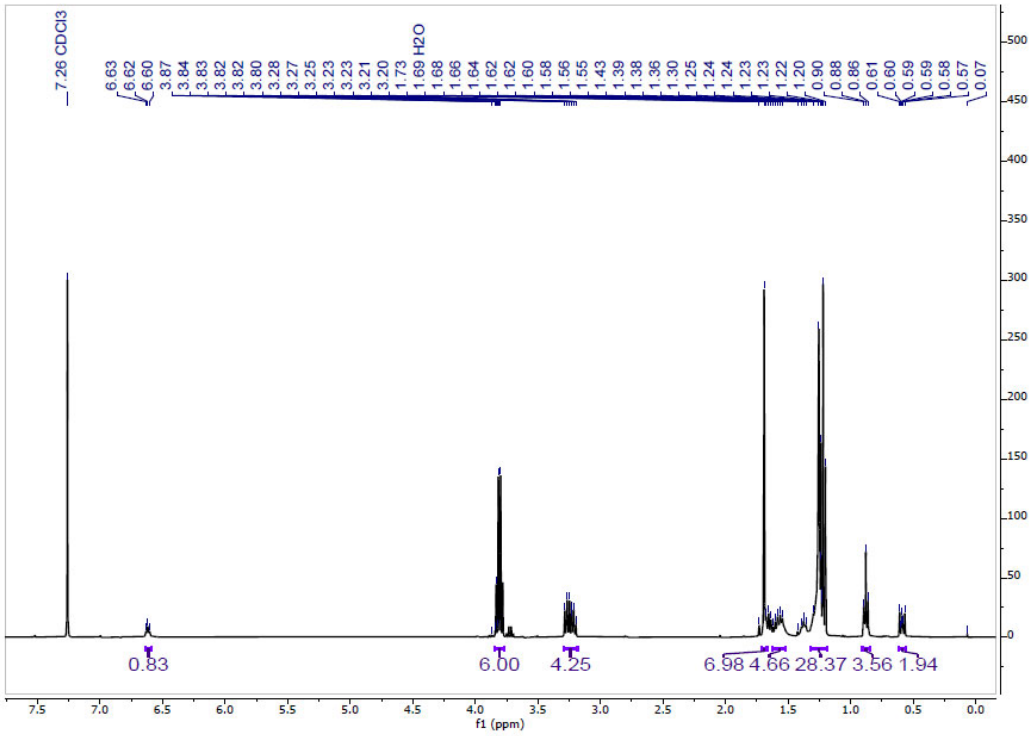

Figure S7. ^{1}H NMR spectrum of **APTES-CTA**.DNA Hybridization Density

3.2. *DNA Hybridization Density*

S-Sil-PNIPAM-DNA or **S-Sulf-PNIPAM-DNA** was introduced into a glass tube, to which 1.5 mL of 4X SSC and 100 µL of a solution of Cy3-labeled DNA solution prepared in PBS 0.1X were added. The slides were incubated at 25°C and 750 rpm for 1h30, then rinsed with MilliQ water and washed twice with 0.1% Tween for 10 minutes (25°C, 750 rpm). After three additional washing cycles with MilliQ water, the glass slides were placed into cleanglass tubes with 2 mL 0.1X PBS, then incubated for 18 min at 85°C and 750 rpm for denaturation. The fluorescence intensity of the supernatants was measured (λ_{exc} = 532 nm and λ_{em} = 568 nm), and the DNA quantity determined using a Cy3-DNA calibration curve in the 1.5-100 nM range. The same procedure was carried out with **S-Sil-PNIPAM-COOH** or **S-Sulf-PNIPAM-COOH** as negative controls. Sequence of the Cy3-tagged complementary reverse can be found in the General Information Section.

3.3. *Water Contact Angle*

After the deposition of a water droplet on **S-Sil-PNIPAM-DNA** or **S-Sulf-PNIPAM-DNA**, the slides were incubated for 3 minutes in a thermoshaker at the target temperature before the measurement of the WCA. All experiments were performed in triplicate.

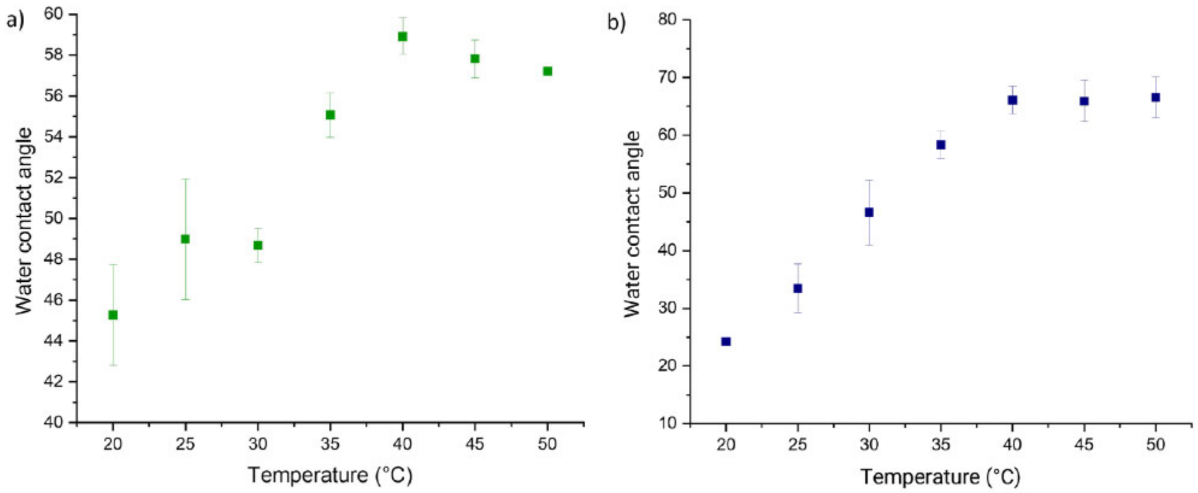

Figure S8. Water contact angle measured at the surface of a) **S-Sil-PNIPAM-DNA** and b) **S-Sulf-PNIPAM-DNA**, from 20°C to 50°C.

3.4. *Protein Fouling Assay*

A total of 10 µL of a solution of BSA-AF488 at 1 mg/mL was deposited on the surfaces, which had been placed at a temperature of 22°C or 42 °C beforehand. The surfaces were then incubated for one hour. The slides were then dipped in PBS (twice, 3 mL) and MiliQ water (3 mL) to remove the weakly bound BSA. The surfaces were then dried and imaged via fluorescence microscopy.
